# Statistical Analysis of Terminal Extensions of Protein **β**-Strand Pairs

**DOI:** 10.1155/2013/909436

**Published:** 2013-01-28

**Authors:** Ning Zhang, Shan Gao, Lei Zhang, Jishou Ruan, Tao Zhang

**Affiliations:** ^1^Department of Biomedical Engineering, Tianjin University, Tianjin Key Lab of BME Measurement, Tianjin 300072, China; ^2^College of Mathematical Sciences and LPKM, Nankai University, Tianjin 300071, China; ^3^College of Life Sciences, Nankai University, Tianjin 300071, China

## Abstract

The long-range interactions, required to the accurate predictions of tertiary structures of **β**-sheet-containing proteins, are still difficult to simulate. To remedy this problem and to facilitate **β**-sheet structure predictions, many efforts have been made by computational methods. However, known efforts on **β**-sheets mainly focus on interresidue contacts or amino acid partners. In this study, to go one step further, we studied **β**-sheets on the strand level, in which a statistical analysis was made on the terminal extensions of paired **β**-strands. In most cases, the two paired **β**-strands have different lengths, and terminal extensions exist. The terminal extensions are the extended part of the paired strands besides the common paired part. However, we found that the best pairing required a terminal alignment, and **β**-strands tend to pair to make bigger common parts. As a result, 96.97%  of **β**-strand pairs have a ratio of 25% of the paired common part to the whole length. Also 94.26% and 95.98%  of **β**-strand pairs have a ratio of 40% of the paired common part to the length of the two **β**-strands, respectively. Interstrand register predictions by searching interacting **β**-strands from several alternative offsets should comply with this rule to reduce the computational searching space to improve the performances of algorithms.

## 1. Introduction

The issue of protein structure prediction is still extremely challenging in bioinformatics [1, 2]. Usually, structural information for protein sequences with no detectable homology to a protein of known structure could be obtained by predicting the arrangement of their secondary structural elements [3]. As we know, the two predominant protein secondary structures are *α*-helices and *β*-sheets. However, a combination of the early suitable *α*-helical model systems and sustained researches have resulted in a detailed understanding of *α*-helix, while comparatively little is known about *β*-sheet [4]. Tertiary structures of *β*-sheet-containing proteins are especially difficult to simulate [3, 5]. Unlike *α*-helices, *β*-sheets are more complex resulting from a combination of two or more disjoint peptide segments, called *β*-strands. Therefore, the *β*-sheet topology is very useful for elucidating protein folding pathways [6, 7] for predicting tertiary structures [3, 8–11], and even for designing new proteins [12–14]. 

As fundamental components, *β*-sheets are plentifully contained in protein domains. In a *β*-sheet, multiple *β*-strands held together linked by hydrogen bonds and can be classified into parallel and antiparallel direction styles. Adjacent *β*-strands bring distant residues on sequences into close special contact with one another and constitute a specific mode of amino acid pairing [1, 15–17], interactions (like DNA base pairing). There is a growing recognition of the importance of the strand-to-strand interactions among *β*-sheets [18]. Several studies, including statistical studies examining frequencies of nearest-neighbor amino acids in *β*-sheets, found a significantly different preference for certain interstrand amino acid pairs at nonhydrogen-bonded and hydrogen-bonded sites [1, 17, 19, 20], Dou et al. [21] created a comprehensive database of interchain *β*-sheet (ICBS) interactions. We also developed the SheetsPair database [22] to compile both the interchain and the intrachain amino acid pairs. 

Generally speaking, previous work on *β*-sheets mainly focused on the interresidue contacts or amino acid partners [23–28]. Prediction of inter-residue contacts in *β*-sheets is interesting, while the prediction by ab initio structure is also useful to understand protein folding [29, 30]. Our previous studies showed that the interstrand amino acid pairs played a significant role to determine the parallel or antiparallel orientation of *β*-strands [15], and the statistical results could possibly be used to predict the *β*-strand orientation [16]. Cheng and Baldi [11] introduced BETAPRO method to predict and assemble *β*-strands into a *β*-sheet, in which a single misprediction of one amino acid pairing from the first stage could be amplified by the next stages and results in serious wrong set of partner assignments between *β*-strands. However, those studies can be viewed as initial steps of *β*-sheet studies relative to predict strand level pairing [25]. In this paper, to go one step further, we investigate the *β*-strand pairing on the strand level for exploring the rules of how *β*-strands form a *β*-sheet.

Many results have shown the importance of statistical analysis in protein structure studies [15, 16]. In particular, statistical information could provide a starting point for de novo computational design methods that are now becoming successful for short, single-chain proteins [14], as well as methods of protein structure predictions and understanding of protein folding mechanisms [31, 32]. Fooks et al. [1] also indicated that such statistical analysis results would be useful for protein structure prediction. Therefore, we advocate using the tools of statistics and informatics to study *β*-sheet and generate new rules for algorithm development. In this study, we focused on the terminal extensions of paired *β*-strands.

## 2. Results

### 2.1. Dataset

All protein structure data used in this study were taken from a PISCES [33, 34] dataset generated on May 16, 2009. In the dataset, the percentage identity cutoff is 25%, the resolution cutoff is 2.0 angstroms, and the *R*-factor cutoff is 0.25. Secondary structures were assigned from the experimentally determined tertiary structures by using the DSSP program. Besides proteins containing disordered regions [35–37], all data were further preprocessed according to the following criteria: (i) no *β*-sheet-containing protein chains were removed; (ii) protein chains with nonstandard three-letter residue names (such as DPN, EFC, ABA, C5C, PLP, etc.) were removed, since these indicate that the protein chains have covalently bounded ligands or modified residues; (iii) protein chains with uncertain structures or incorrect data were removed. Since *β*-bulges tend to be isolated and rare [11], we did not consider *β*-bulges in this study either, as several previous studies did [1, 3]. Finally, 2,315 protein chains were extracted, containing 19,214 *β*-strand pairs. Note that in the special case of *β*-bulges, no amino acid pair is assigned.

### 2.2. The *β*-Sheet Structure

**Figure 1:**
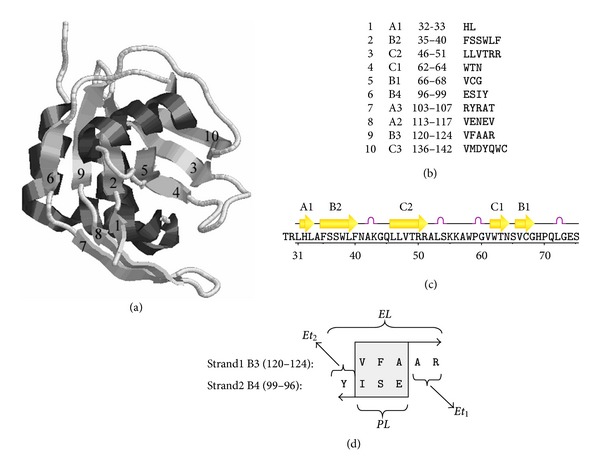
An illustrated example of *β*-strand pairing in a *β*-sheet (PDB code: 1HZT). (a) The sketch of the tertiary structure of the protein produced by using RASMOL. Protein 1HZT is an *α*/*β* protein with 10 *β*-strands numbered from 1 to 10, forming seven different strand pairs. (b) The sequences of the 10 *β*-strands with their initial and ending residue numbers. (c) The 10 *β*-strands in the linear primary sequence. (d) An example of a *β*-strand partnership graph. The pairing is between strands “B3” and “B4,” with the light gray box representing the common pairing part.

The *β*-sheets, where two or more *β*-strands are arranged in a specific conformation, are illustrated in Figure 1(a), by a protein example (PDB code 1HZT). Adjacent strands, or the so-called strand pairs, can either run in the same (parallel) or in the opposite (antiparallel) direction styles. In protein 1HZT, there are 3 *β*-sheets called A, B, and C, formed by 10 different *β*-strands numbered from 1 to 10, making 7 different *β*-strand pairs, respectively. The 10 *β*-strands can be named by the *β*-sheet each belongs to and the index numbers in the order of partnership. For example, the 3 *β*-strands forming *β*-sheet A can be called “A1,” “A2,” and “A3,” while other 4 *β*-strands forming *β*-sheet B can be called “B1,” “B2,” “B3,” and “B4,” respectively. “A1-A2,” “A2-A3,” “B1-B2,” “B2-B3,” and “B3-B4” are all *β*-strand pairs. Sequences of the 10 *β*-strands with their initial and ending residue numbers are also given in Figure 1(b).

### 2.3. Different Lengths of Paired *β*-Strands

**Figure 2:**
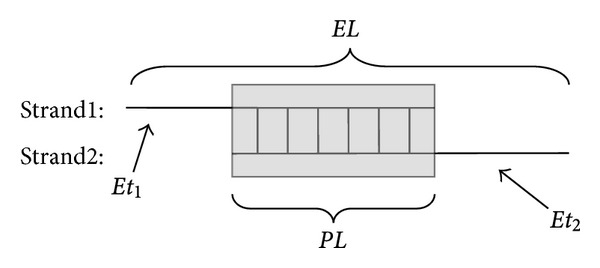
A schematic diagram of terminal extensions of *β*-strand pairs. The two blank lines represent the two *β*-strands, respectively. The light gray box represents the common pairing part of the two *β*-strands with amino acid pairing.

For a *β*-strand pair, the terminal of one *β*-strand does not always align with the terminal of the other (Figure 2), making “terminal extensions” besides the common paired parts. Note that only amino acids in the common part construct amino acid pairs. 

Why “terminal extensions” exist widely in *β*-strand pairs? We firstly investigated the lengths of two paired *β*-strands and then calculated the percent of each case whether the “terminal extensions” exist or not. Results are shown in Table 1. 

As shown in Table 1, the two paired *β*-strands having the same length only account for 29.53% of all samples. In other 70.47% percent of samples, lengths of the two paired *β*-strands are different. 

### 2.4. Statistical Results of Variables

We define the following variables.

1. Let *SL*_1_ and *SL*_2_ represent the lengths of two paired *β*-strands, respectively. Length of the *β*-strand with smaller strand number (strand numbers can be obtained from PDB database) is defined as *SL*_1_, while length of the other *β*-strand is defined as *SL*_2_.
2. Let *PL* stand for the length of the common part, which is often smaller than *SL*_1_ and *SL*_2_.
3. Terminal extensions can be found in either of the two *β*-strands. We define the lengths of the two terminal extensions *Et*_1_ and *Et*_2_, respectively. Length of the terminal extension of the *β*-strand with length *SL*_1_ is defined as *Et*_1_while the other as *Et*_2_.
4. Let *EL* represent the whole length; *EL* = *PL* + *Et*_1_ + *Et*_2_.

Then, the paring ratio *R* could be calculated by

(1) $$\begin{array}{c} R=\frac{PL}{EL}\times 100\%=\frac{PL}{PL+Et_{1}+Et_{2}}\times 100\%. \end{array}$$

The ratio of the common paired part to the length of each *β*-strand (*i* = 1,2) could be calculated by

(2) $$\begin{array}{c} Rt_{i}=\frac{PL}{SL_{i}}\times 100\%,\;i=1,2. \end{array}$$

A small percent of *β*-strand pairs have no “terminal extensions,” the *R*, *Rt*_1_, and *Rt*_2_ values for which will be 100%. 

We calculated *PL*, *Et*_1_, *Et*_2_, *EL* for all *β*-strand pairs in the present dataset.  Table 2 gives the range of these variables as well as the averages and standard deviations. 

**Figure 3:**
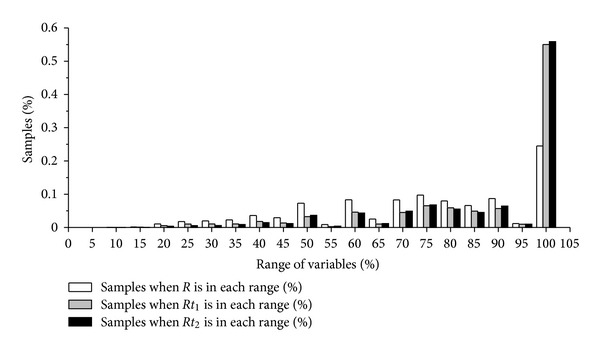
Distribution of *R*, *Rt*_1_, and *Rt*_2_ variables in the current dataset.

We also calculated *R*, *Rt*_1_, and *Rt*_2_ for all *β*-strand pairs in the present dataset. The distribution of these variables is shown in Figure 3. 

## 3. Discussion

### 3.1. Strands Tend to Align Their Terminals

For the 70.47% of samples with different strand lengths, although they have different lengths, the differences are not big for most of them. Only a small percent of samples (below 2.09%) have the difference above 5. In these cases, it is obvious that they cannot align the terminals (with both *Et*_1_ = 0 and *Et*_2_ = 0). They have two ways to choose from: either align to only one terminal making another “terminal extension”, or align to none of the two terminals making both “terminal extensions.” However, it can be seen from Table 1 that most *β*-strands tend to be in the former case. For example, in case of the length difference 1, the former case accounts for 85.18% while the latter only 14.82%. It is consistent with the case of same-length strand pairs, in which *β*-strands tend to align their terminals with each other. Interestingly, it is suggested that *β*-strands tend to align their terminals. In different-length strand pairs, they still retain one terminal alignment, although they can not align both ends. 

### 3.2. Small “Terminal Extensions”

From Table 2, it can be seen that lengths of *β*-strands are not very long, ranging from 1 to 25 with an average length about 4-5 amino acids. The averages and the standard deviations are similar between lengths of the two paired *β*-strands (*SL*_1_ and *SL*_2_). 

The length of the common part *PL* has a range similar to that of lengths of *β*-strands. This indicates that although “terminal extensions” exist, common pairing parts occupy most of *β*-strands, while “terminal extensions” occupy least. The fact that the maximum value of *EL* is 29, only a little bigger than that of lengths of *β*-strands, and the fact that in average both the “terminal extensions” only have about 1 amino acid (*Et*_1_ = 1.05 and *Et*_2_ = 1.03) also support this assumption. 

Figure 3 gives percent of samples for *R*, *Rt*_1_, and *Rt*_2_ in each range of their possible values (from 0% to 100%), respectively. It can be seen that the distributions of *Rt*_1_ and *Rt*_2_ are similar. More than half of the *β*-strand pairs have these two variables above 95% (or in the range (95–100)). Big *Rt*_1_ or *Rt*_2_ means big common part of *β*-strands, or small “terminal extensions.” Rare *β*-strand pairs have smaller values of *R*, *Rt*_1_, and *Rt*_2_, which indicates that most *β*-strands do not pair by means of small “common part” or big “terminal extensions.” It could be concluded from the results that *β*-strands tend to pair with bigger pairing common parts, leaving smaller “terminal extensions.”

### 3.3. Possible Reasons for *β*-Strand Extensions

Why “terminal extensions” exist so widely in *β*-strand pairs? The fact that lengths of two paired *β*-strands are not the same in most cases as shown in Table 1 may be one of the possible reasons. If paired *β*-strands have the same lengths, most of them (82.95%) tend to align their terminals with each other, leaving no “terminal extensions.” 

A *β*-strand is led to pair with another by several kinds of potential forces. Steward and Thornton [3] indicated that a single *β*-strand was still able to recognize a noninteracting *β*-strand with greater accuracy than that in the case of between two random sequences. The potential forces include hydrogen bonds, van der Waals forces, electrostatic interaction, ionic bonds, hydrophobic effects, and so forth. Parisien and Major [38] revealed that among all the forces, the most important one was the construction of a hydrophobic face. It is conceivable that one residue of a *β*-strand prefers to pair with the residue of another resulting in a stable state of hydrophobic effects. Optimizing such interactions may result in extensions, which could be the second reason, since more often than not the “terminal alignment” is not the case of optimized pairing style.

A third possible reason could be due to the nucleation events that initiate the *β*-sheet folding. Amino acids in the central part could pair firstly and then fold to extend to terminals. 

Another reason is the roles of the nonpaired terminal amino acids in stabilizing the *β*-sheet structure. Several other studies have identified their key roles in modulating protein folding rates, stability, and folding mechanism [39–43]. Therefore, the *β*-strand terminals could also be important factors for a *β*-sheet formation. 

### 3.4. Ratio Rule of Pairing Strand Alignment

**Figure 4:**
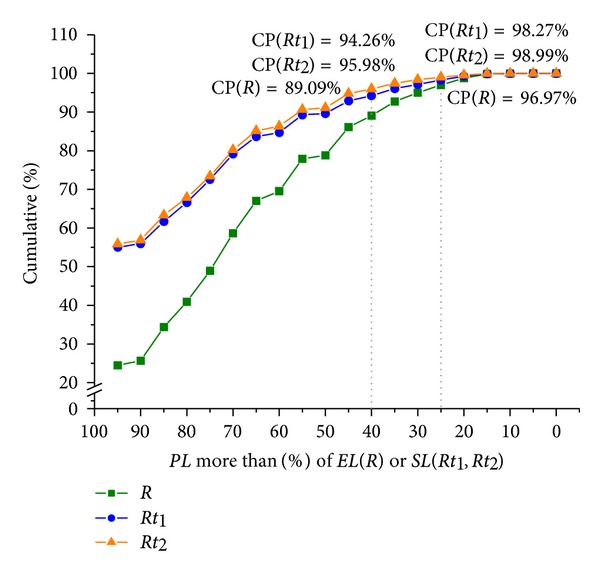
Cumulative percentages (CPs) of *R*, *Rt*_1_, and *Rt*_2_ calculated from the present dataset. The horizontal axis denotes the percentage of common paired region *PL* to *EL* (for curve *R*) or to *SL* (for curves *Rt*_1_ and *Rt*_2_). Points on the *R* curve denote the cumulative percentages of samples whose *R* = *PL*/*EL* equals or is bigger than the corresponding abscissa value. Points on the *Rt*_1_ and *Rt*_2_ curves denote the cumulative percentages of samples whose *Rt*_1_ = *PL*/*Rt*_1_ or *Rt*_2_ = *PL*/*Rt*_2_ equals or is bigger than the corresponding abscissa value, respectively.

To quantify the pairing common part of paired *β*-strands, we calculated the cumulative percent of variables *R*, *Rt*_1_, and *Rt*_2_ and depicted them in Figure 4.

From Figure 4, it can be seen that when *Rt*_1_ ≥ 40% and *Rt*_2_ ≥ 40%, the cumulative percentages reach 94.26% and 95.98%, respectively, while when *R* ≥ 40% only 89.89%. When *R* ≥ 25%, the cumulative percentages reach up to 96.97%. Therefore, a rule can be made of the alignment of *β*-strand pair as follows:

(3) $$\begin{array}{c} R\geq 25\%,\;\;{Rt}_{i}\geq 40\%,\;i=1,2. \end{array}$$

Almost all samples (above 94%) obey this rule. 

In a *β*-strand alignment prediction algorithm, all possible pairings should be examined and scored; it is a time-consuming task. Kato et al. [44] stated that prediction of planar *β*-sheet structures was NP-hard in the present state of our knowledge (http://en.wikipedia.org/wiki/NP-hard). However, this previous rule should be used as a constraint of the relative positions in *β*-strand alignment to reduce the computational searching space, which could be used to develop high-speed *β*-strand topology prediction algorithms.

## 4. Conclusion

At the most straightforward level, full “identification” of a *β*-strand pair could consist of (i) finding the interacting partner *β*-strand(s), (ii) predicting the relative orientation (i.e. parallel or antiparallel), and (iii) shifting the relative positions of the two interacting *β*-strands [15, 16]. In this study, we focused on the third aspect. The formation of protein structure and protein folding mechanism are very complex, and the mechanisms of *β*-sheet formation are unclear [45]. However, simple rules could contribute to developing new algorithms in the step of full prediction of *β*-sheet and understanding of protein folding pathways in ongoing research. 

In this study, to go one step further, we studied *β*-sheets on the strand level instead of amino acid level. Statistical analyses of the terminal extensions of paired *β*-strands were performed and a simple rule “*R* ≥ 25% and *Rt*_*i*_ ≥ 40%, *i* = 1,2” was made. Steward and Thornton [3] developed an information theory approach to predict the relative offset positions by shifting one *β*-strand up to 10 residues either side of that observed. Such a rule could be used in similar studies. We certainly believe that the conclusions presented in this study could contribute to predict protein structures and to develop *β*-sheet prediction methods.

## Figures and Tables

**Table 1:** Statistical results of lengths of two paired *β*-strands and percent of samples in each case whether the two “terminal extensions” exist or not.

Abs (*SL*_1_ − *SL*_2_)*	Number of pairs	Percent	Percent of *Et*_1_ = 0 and *Et*_2_ = 0	Percent of *Et*_1_ = 0 and *Et*_2_ > 0	Percent of *Et*_1_ > 0 and *Et*_2_ = 0	Percent of *Et*_1_ > 0 and *Et*_2_ > 0
0	5673	29.53%	82.95%	0.00%	0.00%	17.05%
1	5633	29.32%	0.00%	40.48%	44.70%	14.82%
2	3170	16.50%	0.00%	30.57%	41.10%	28.33%
3	1798	9.36%	0.00%	28.59%	31.15%	40.27%
4	1016	5.29%	0.00%	26.77%	29.82%	43.41%
5	618	3.22%	0.00%	29.13%	27.51%	43.37%
6	401	2.09%	0.00%	30.42%	25.69%	43.89%
7	323	1.68%	0.00%	25.39%	32.51%	42.11%
8	247	1.29%	0.00%	16.19%	30.36%	53.44%
9	101	0.53%	0.00%	26.73%	24.75%	48.51%
10	69	0.36%	0.00%	20.29%	39.13%	40.58%
>10	165	0.86%	0.00%	15.15%	27.27%	57.58%

*Absolute value of the difference of *SL*_1_  −  *SL*_2_.

**Table 2:** Statistical results of variables of *β*-strand pairs in the current dataset.

	Minimum value	Maximum value	Average	Standard deviation
*SL*_1_	1	25	4.99	2.82
*SL*_2_	1	25	4.90	2.80
*PL*	1	23	4.86	2.26
*Et*_1_	0	18	1.15	1.79
*Et*_2_	0	22	1.03	1.64
*EL*	2	29	7.03	3.09
